# Gravity-Sensing Tissues for Gravitropism Are Required for “Anti-Gravitropic” Phenotypes of *lzy* Multiple Mutants in Arabidopsis

**DOI:** 10.3390/plants9050615

**Published:** 2020-05-12

**Authors:** Nozomi Kawamoto, Yuta Kanbe, Moritaka Nakamura, Akiko Mori, Miyo Terao Morita

**Affiliations:** 1 Division of Plant Environmental Responses, National Institute for Basic Biology, Myodaiji, Okazaki 444–8556, Japan; kawamoto@hhu.de (N.K.); m-nakamu@nibb.ac.jp (M.N.); 2 Graduate School of Bioagricultural Sciences, Nagoya University, Furocho, Chikusa, Nagoya 464–8601, Japan; betayuta02@gmail.com (Y.K.); beaker1016@gmail.com (A.M.)

**Keywords:** gravitropism, *LAZY1-LIKE*, GSA, AGO, columella, endodermis

## Abstract

Plant posture is controlled by various environmental cues, such as light, temperature, and gravity. The overall architecture is determined by the growth angles of lateral organs, such as roots and branches. The branch growth angle affected by gravity is known as the gravitropic setpoint angle (GSA), and it has been proposed that the GSA is determined by balancing two opposing growth components: gravitropism and anti-gravitropic offset (AGO). The molecular mechanisms underlying gravitropism have been studied extensively, but little is known about the nature of the AGO. Recent studies reported the importance of *LAZY1-LIKE* (*LZY*) family genes in the signaling process for gravitropism, such that loss-of-function mutants of *LZY* family genes resulted in reversed gravitropism, which we term it here as the “anti-gravitropic” phenotype. We assume that this peculiar phenotype manifests as the AGO due to the loss of gravitropism, we characterized the “anti-gravitropic” phenotype of *Arabidopsis* *lzy* multiple mutant genetically and physiologically. Our genetic interaction analyses strongly suggested that gravity-sensing cells are required for the “anti-gravitropic” phenotype in roots and lateral branches. We also show that starch-filled amyloplasts play a significant role in the “anti-gravitropic” phenotype, especially in the root of the *lzy* multiple mutant.

## 1. Introduction

Plants use various signals, such as light and gravity signals, to optimize shoot and root architecture to achieve directional organ growth. Previous studies have suggested that amyloplasts, plastids that accumulate starch, move in the direction of gravity to trigger a signaling process, known as gravitropism [1,2,3]. This starch-statolith hypothesis is supported by the reduced gravitropism of the starchless *phosphoglucomutase* (*pgm*) mutant [1,3,4]. The cell ablation studies demonstrated that root cap columella cells containing sedimentable amyloplasts are important for root gravitropism [5,6]. Thus, columella cells are considered to be major gravity sensing cells in roots. Meanwhile, additional genetic studies with a series of *shoot gravitropism* (*sgr*) mutants provided evidence for gravity-sensing cells in the *Arabidopsis* shoot [7]. It has been reported that *sgr1* (also known as *scarecrow* (*scr*)) and *sgr7* (also known as *short-root* (*shr*)) are agravitropic in the shoot, and these mutants lack endodermal cell layers in inflorescence stems [8]. The endodermal cells in *Arabidopsis* shoots contain amyloplasts that can fall due to gravity. Furthermore, the *endodermal-amyloplast less 1* (*eal1*) mutant was reported as an agravitropic mutant in inflorescence stem [9]. The *eal1* was mapped on the At4g37650 locus which is also known as *SHR* locus. The *eal1* carries three nucleotides deletion which correspond to glutamate 230 and results in a single amino acid deletion of SHR [10]. The *eal1* develops endodermal-like tissues without gravity sensing ability [10], while the *sgr7* completely loses endodermis [8]. Thus, the *eal1* is thought to be a hypomorphic mutant allele of *sgr7*/*shr*. These results indicate that endodermal cells are gravity-sensing cells in the Arabidopsis shoot [11].

Recently, key genes for gravitropism, including *LAZY1* and its orthologous *LAZY1-LIKE* (*LZY*) family genes, were identified in rice and *Arabidopsis* [12,13]. The loss-of-function mutants of *LAZY1* in rice and *LZY1* in *Arabidopsis* resulted in reduced shoot gravitropism [12,13]. In *Arabidopsis*, *LZY1*, *LZY2*, and *LZY3* genes are expressed in the endodermis and function redundantly in shoot gravitropism [14,15]. LZY2 and LZY3 are localized to the plasma membrane, while LZY1 is localized to the nucleus and plasma membrane [13,14,15]. Although a mutated LZY1 does not localize in the nucleus, it remains associated with shoot gravitropism, suggesting that plasma membrane-localized LZY1 is functionally important for gravitropism [13]. In root, LZY2, LZY3, and LZY4 are expressed in columella cells and function redundantly in gravitropism [14,15,16]. Recently, we reported that LZY3 is localized to the plasma membrane and changed its polar localization in response to gravi-stimulation by reorienting its roots [17]. It has been suggested that polar localized LZY3 recruits RCC1-LIKE DOMAIN (RLD) proteins to control polar auxin transport via auxin efflux carrier PIN proteins [17]. Taken together, LZY proteins likely play a central role in gravity-sensing cells to connect signals from physical amyloplast sedimentation to polar auxin transport. Asymmetrically distributed auxin then induces differential organ growth to achieve the gravitropic response.

Lateral roots and shoot branches maintain specific growth angles relative to the direction of gravity, which is known as the gravitropic setpoint angle (GSA). It has been proposed that the GSA is determined by a balance between gravitropism and anti-gravitropic offset (AGO), which is a growth component that counteracts gravitropic growth [18]. Physiological and genetic analyses suggest that auxin signaling, transport, and its regulatory control of gene expression are all involved in AGO and gravitropism [19,20]. In addition, *sgr5* was identified as a gravitropism-deficient mutant, and its lateral branches tend to grow horizontally [21]. The *SGR5* gene encodes a C2H2-type zinc finger protein, and it has been demonstrated that SGR5/INDETERMINATE DOMAIN15 (IDD15), IDD14, and IDD16 control auxin distribution via transcriptional control of auxin biosynthetic genes and the auxin transporter *PIN1* [21,22]. Loss-of-function mutants of *LZY* genes have reduced gravitropism, and their shoot branches and lateral roots tend to grow horizontally [13,14,15]. Therefore, LZY family proteins appear to contribute to GSA control through gravity signaling that regulates polar auxin transport. However, in contrast to this understanding of the molecular mechanism underlying gravitropism, little is known about the mechanisms controlling AGO.

It has been reported that *LZY* family genes control growth direction of the primary root in *Medicago truncatula* and *Arabidopsis* [14,16,23]. Loss of function of three *LZY* genes, namely, *LZY2*, *LZY3*, and *LZY4*, reverses the growth direction of primary roots in response to gravi-stimulation [14,16,23]. This phenotype is termed “negative gravitropism” [14,16,23], but the “negative gravitropic” phenotype is not simply a mirror image of the normal positive gravitropism. Rather, the responsiveness to gravi-stimulation of primary roots of the *lzy* triple mutant is considerably weak [14]. Meanwhile, we reported that primary shoots of the *lzy1;2;3* triple mutant are agravitropic, while its lateral branches curl downward [15]. We refer to this phenotype of reversed growth direction observed in both the primary root of the *lzy2;3;4* mutant and the lateral branches of the *lzy1;2;3* mutant as “anti-gravitropic.” Therefore, with respect to GSA, both “anti-gravitropic” phenotypes of primary roots and lateral branches lead us to hypothesize that the loss of *LZY* function results in manifestation of AGO [24]. 

In this study, we characterized the “anti-gravitropic” phenotype by analyzing *lzy* multiple mutants and gravitropism-deficient mutants in primary roots and lateral branches. Our genetic and physiological studies highlight the importance of columella and endodermal cells in roots and shoots for GSA determination. In this process, we demonstrated that starch-accumulated amyloplasts in columella cells are required for the recognition of the gravity vector to achieve both gravitropism and “anti-gravitropic” phenotypes in primary root. In shoots, while there is a marginal contribution of amyloplasts, endodermal cells remain functionally important for gravitropism and “anti-gravitropic” phenotypes.

## 2. Results

### 2.1. Amyloplast Sedimentation Leads to Directional Growth of the Primary Root

**Figure 1:**
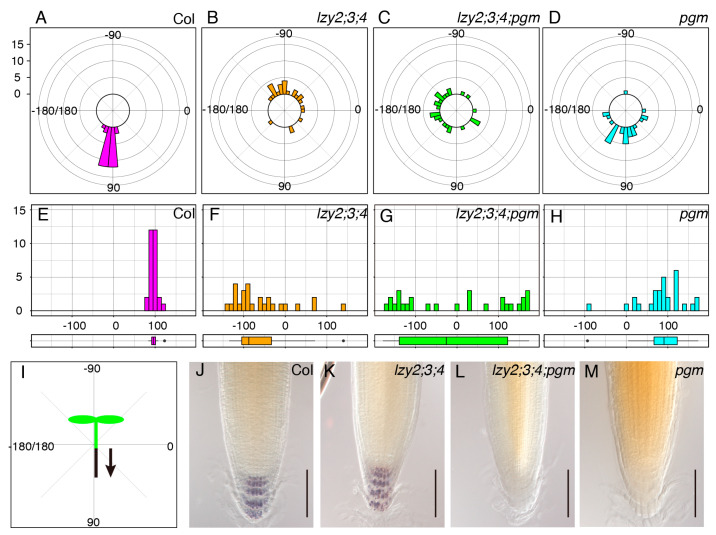
Genetic analysis of growth direction of primary roots. Polar distribution of growth direction of primary root in wild-type Col (**A**), *lzy2;3;4* (**B**), *lzy2;3;4;pgm* (**C**), and *pgm* (**D**). Growth angles were plotted on histograms and distributions were evaluated with boxplots from wild-type Col (**E**), *lzy2;3;4* (**F**), *lzy2;3;4;pgm* (**G**), and *pgm* (**H**). Schematic representation of growth angle quantification (**I**). Each bar indicates the number of roots. Lugoal’s staining of primary roots of wild-type Col (**J**), *lzy2;3;4* (**K**), *lzy2;3;4;pgm* (**L**), and *pgm* (**M**) at 7 days after transferring to phytocabinet. Bars indicate 100 µm.

We assessed the growth direction of primary roots of 5-day-old seedlings on a vertically standing plate (Figure 1). Wild-type roots grew in the direction of gravity, but roots of the *lzy2;3;4* triple mutant tended to grow upward in an “anti-gravitropic” direction, as previously reported [14,23] (Figure 1A,B,E,F,I). For gravitropism, amyloplast sedimentation in gravity-sensing cells is important to recognize the direction of gravity [2,11]. The *pgm* mutant is starch synthesis deficient with “light” starchless amyloplasts and partially complete root gravitropism (Figure 1D,H) [4]. To examine whether amyloplasts that accumulate starch were important for the “anti-gravitropic” phenotype of the *lzy2;3;4* triple mutant, we analyzed the genetic interaction between *lzy2;3;4* and *pgm* by generating the *lzy2;3;4; pgm* quadruple mutant and testing the growth direction of primary roots. Since *pgm* carries a mutation in the *phosphoglucomutase* gene which is involved in starch biosynthesis and results in starchless “light” amyloplasts development, it is an ideal mutant to investigate the contribution of starch-filled amyloplasts to “anti-gravitropic” phenotype. Interestingly, directional growth against gravity was eliminated by adding the *pgm* mutation in *lzy2;3;4* plants (Figure 1C,G). As reported previously, starch accumulation was not observed in *pgm* and *lzy2;3;4;pgm* (Figure 1L,M), while wild-type and *lzy2;3;4* accumulated starch (Figure 1J,K). These results demonstrate that *pgm* mutation is epistatic to the *lzy2;3;4* mutant, and starch accumulation in amyloplasts is necessary for the “anti-gravitropic” phenotype of the *lzy2;3;4*. Therefore, gravitropic and “anti-gravitropic” growth is likely to share a similar gravity-sensing mechanism in primary roots.

### 2.2. Lateral Branch Responses to Gravi-Stimulation in the lzy1;2;3 Mutant

**Figure 2:**
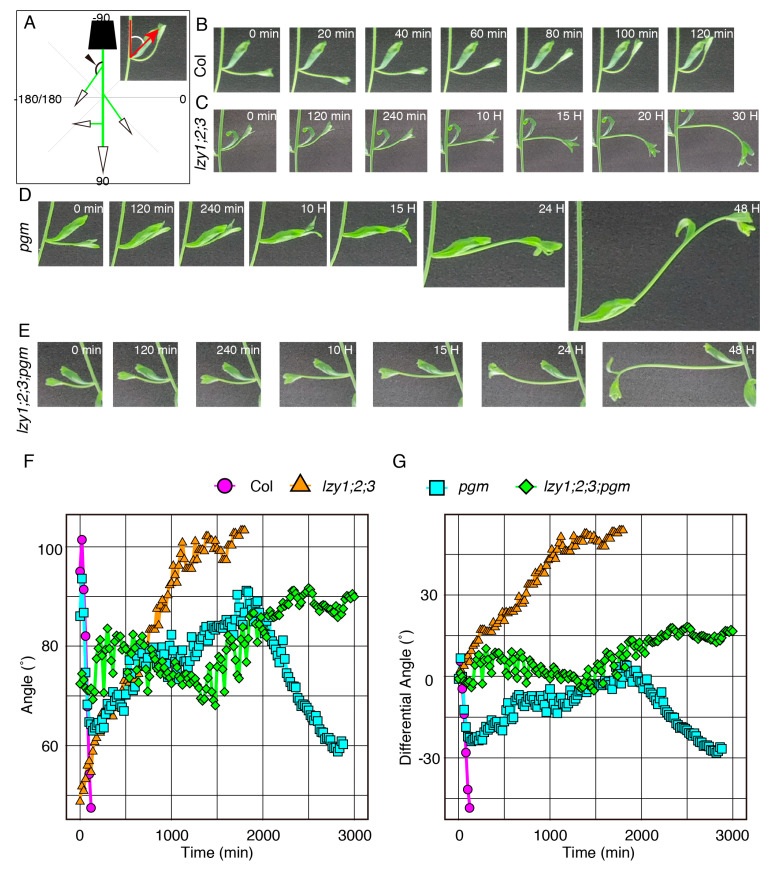
The gravitropic responses of lateral branches. (**A**) Graphical image of the quantification of growth angles of lateral branches. Time-lapse images of lateral branches from wild-type Col (**B**), *lzy1;2;3* (**C**), *pgm* (**D**), *lzy1;2;3;pgm* (**E**) plants after inversion. Immediately after inverting plants, images were obtained at 5 min intervals. Quantification of growth posture change of lateral branches during the gravitropic responses (**F**) and differences of growth angle from initial angles (**G**).

As reported previously, the *lzy1;2;3* triple mutant displayed severe defects in gravitropism in the primary shoot and downward curling of lateral branches [14,15]. However, it remains unclear whether the downward-curling phenotype of the lateral branch is the result of a response to gravi-stimulation or simple epinastic growth. To address this question, we inverted 4-week-old plants containing 1.5–2 cm lateral branches and measured the growth angle of lateral branches (Figure 2A). In wild-type plants, lateral branches began growing upward within 60 min after inversion and completely changed their posture within 120 min (Figure 2B,F,G, Video S1). By contrast, the lateral branches of *lzy1;2;3* gradually grew downward after inversion, and it took considerably longer to complete the posture change (Figure 2C,F,G, Video S2). This result indicates that the lateral branches of *lzy1;2;3* respond to gravi-stimulation and exhibit the “anti-gravitropic” phenotype. This result also suggests that the loss of function of *LZYs* resulted in the “anti-gravitropic” phenotype in lateral branches, resulting in the downward curling of branches.

### 2.3. Endodermal Cells are Required for the “Anti-Gravitropic” Phenotype in Shoots

**Figure 3:**
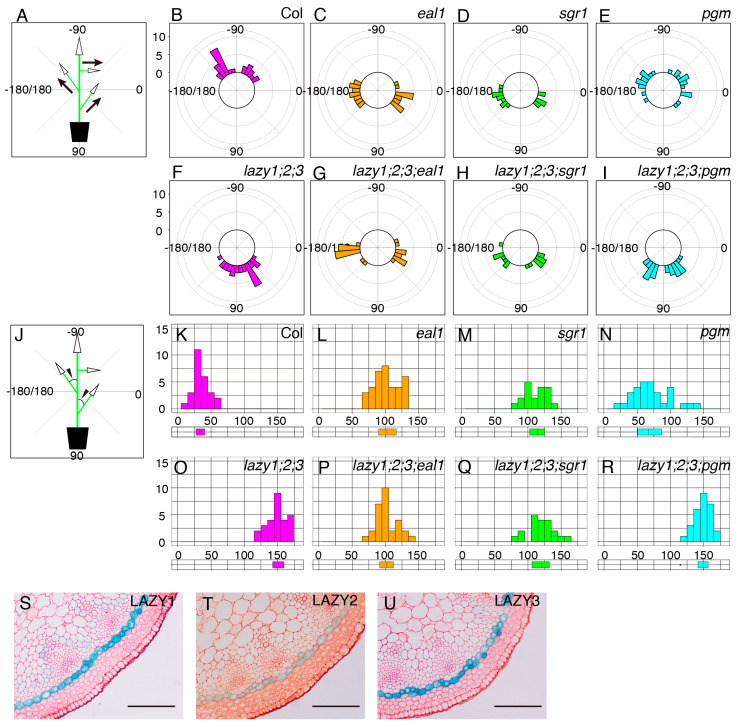
Growth angles of lateral branches. Schematic representation of growth angle evaluation for posture of lateral branches (**A**) and differences from the vertical main axes (**J**). Growth angles of lateral branches from wild-type Col (**B**), *eal1* (**C**), *sgr1* (**D**), *pgm* (**E**), *lzy1;2;3* (**F**), *lzy1;2;3;eal1* (**G**), *lzy1;2;3;sgr1* (**H**), and *lzy1;2;3;pgm* (**I**). Distribution of differential growth angles from the primary flower stem from wild-type Col (**K**), *eal1* (**L**), *sgr1* (**M**), *pgm* (N), *lzy1;2;3* (**O**), *lzy1;2;3;eal1* (**P**), *lzy1;2;3;sgr1* (**Q**), and *lzy1;2;3;pgm* (**R**). Distributions were evaluated as boxplots. Expression patterns of *LZY1*, *LZY2*, and *LZY3* in lateral branches. GUS staining of lateral branches expressing *pLZY1:GUS* (**S**), *pLZY2:GUS* (**T**), and *pLZY3:GUS* (**U**). Bars indicate 100 µm.

In addition to the roots, amyloplast sedimentation is thought to be important for gravity sensing in shoot gravitropism [2,9]. Therefore, we examined whether starch-accumulated amyloplasts were required for the “anti-gravitropic” phenotype of lateral branches in the *lzy1;2;3* mutant. First, using the *lzy1;2;3;pgm* quadruple mutant, we analyzed the lateral branch phenotype. When lateral branches reached 10 cm, plants were photographed and growth angles of axillary branches were calculated (Figure 3A). Differences from vertical axis were calculated as growth angles and plotted as a histogram (Figure 3J). Lateral branches of wild-type plants tended to grow at a 30° angle (Figure 3B,K), while those of *lzy1;2;3* mutants grew at an angle of around 150° (Figure 3F,O). The growth angle of lateral branches of the *pgm* mutant was significantly wider than the wild type, but the phenotype was much weaker than that of the *lzy1;2;3* mutant (Figure 3E,N). Based on our results from the primary roots, we expected that *pgm* also would be epistatic to *lzy1;2;3* in lateral branches. However, this does not appear to be the case with regard to the “anti-gravitropic” phenotype of lateral branches (Figure 3E,F,I,N,O,R).

To investigate the contribution of *pgm* in more detail, we examined the gravitropic response of *pgm* and *lzy1;2;3;pgm* plants upon gravi-stimulation by reorientation. Lateral branches of the *pgm* mutant were able to respond to a new gravity vector, but it took longer to complete the gravitropic response (Figure 2D,F,G and Video S3). Although no clear difference was observed in the growth angles of lateral branches between *lzy1;2;3* and *lzy1;2;3;pgm* under normal growth conditions (Figure 3E,F,I,N,O,R), the *pgm* mutation delayed the “anti-gravitropic” response in *lzy1;2;3* plants upon gravi-stimulation (Figure 2E–G and Video S4). Even if the *pgm* has a minor impact on the lateral branch phenotype of *lzy1;2;3* plants, our results suggest that *pgm* is an epistatic mutation to *lzy1;2;3*.

**Figure 4:**
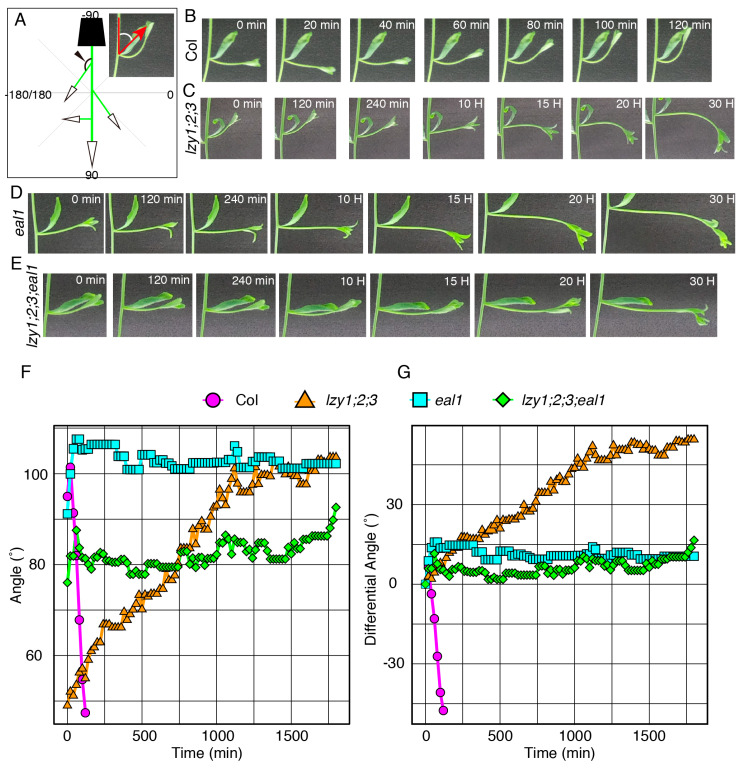
The gravitropic responses of lateral branches. (**A**) Graphical image of the quantification of growth angles of lateral branches. Time-lapse images of lateral branches from wild-type Col (**B**), *lzy1;2;3* (**C**), *eal1* (**D**), *lzy1;2;3;eal1* (**E**) plants after inversion. Immediately after inverting plants, images were obtained at 5 min intervals. Quantification of growth posture change of lateral branches during the gravitropic responses (**F**) and differences of growth angle from initial angles (**G**). Images of Col and *lzy1;2;3* are identical to Figure 2.

We then investigated the relationship between the endodermis and the “anti-gravitropic” phenotype in *lzy1;2;3* plants. Endodermal cells where *LZY* genes are expressed and function are gravity-sensing cells for shoot gravitropism [8]. As has been shown in primary shoots [15], promoter activity of *LZY1*, *LZY2*, and *LZY3* were detected in the endodermis of lateral branches (Figure 3S–U) and affect the growth angle phenotype. Two key transcription factors, SCR and SHR, function during development of the endodermis in *Arabidopsis* [25,26,27,28]. *LZY* genes were downregulated in the *eal1* mutant, a hypomorphic allele of *sgr7/shr*, as well as in *sgr1/scr* [15]. The *eal1* mutant expressed SHR with an amino acid deletion form endodermal-like tissue which lacked the ability as a gravity-sensing tissue [9,10]. In contrast to *eal1*, *sgr1* completely lost its endodermis in the inflorescence stem [8]. Both endodermis-deficient mutants display almost completely horizontal lateral branches, with 100°–110° growth angles (Figure 3C,D,L,M). To investigate their genetic relationship, we generated quadruple mutants, *lzy1;2;3;eal1* and *lzy1;2;3;sgr1*, and analyzed the growth angles of the lateral branches. The downward-curling phenotype observed in the *lzy1;2;3* mutant was clearly suppressed by *eal1* and *sgr1*, resulting in straight lateral branches growing horizontally (Figure 3G,H,P,Q). Moreover, lateral branches of *eal1* and *lzy1;2;3;eal1* plants failed to respond to gravi-stimulation by inversion (Figure 4D–G, Supplementary Movie S5,6). While *eal1* and *lzy1;2;3;eal1* failed to develop endodermal tissues, they developed properly in lateral branches of wild-type and *lzy1;2;3* plants. Taken together, the endodermal tissue appears crucial for the normal gravitropism and “anti-gravitropic” phenotypes of lateral branches in *lzy1;2;3* triple mutants, but it remains unclear whether gravitropism and “anti-gravitropic” phenotypes share a similar gravity-sensing mechanism.

### 2.4. Suppressor Screening of lzy2;3;4 in Primary Roots

**Figure 5:**
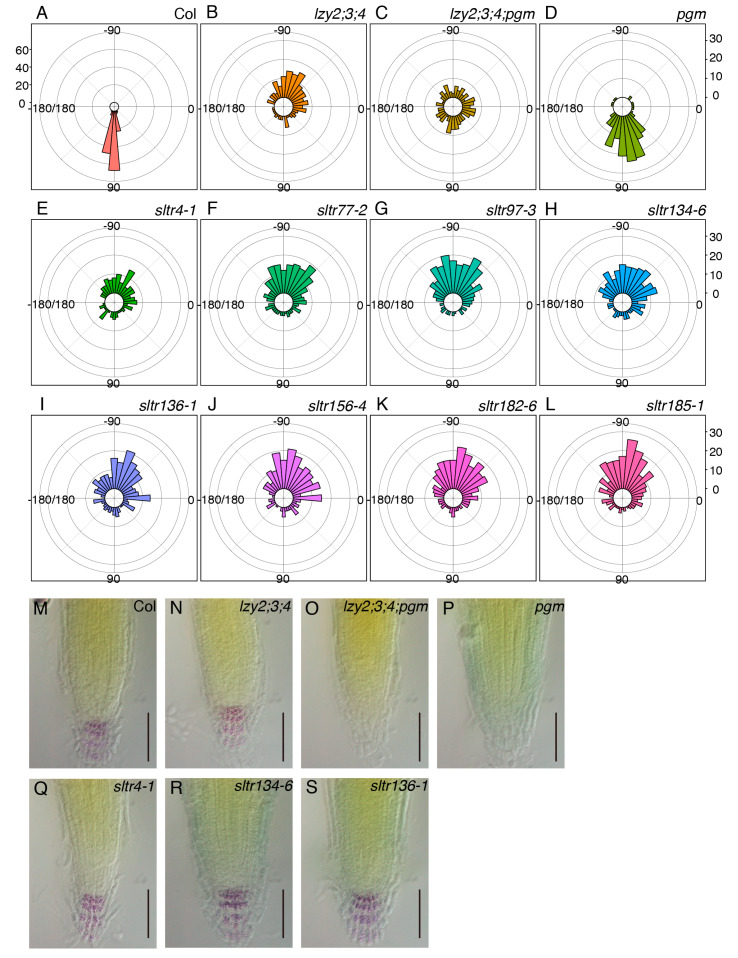
Genetic screening of *lzy2;3;4* suppressors. Growth direction of primary roots in wild-type Col (**A**), *lzy2;3;4* (**B**), *lzy2;3;4;pgm* (**C**), and *pgm* (**D**) and *sltr* candidates, *sltr4-1* (**E**), *sltr77-2* (**F**), *sltr97-3* (**G**), *sltr134-6* (**H**), *sltr136-1* (**I**), *sltr156-4* (**J**), *sltr182-6* (**K**), and *sltr185-1* (**L**). More than 150 plants were evaluated for all genotypes. Bars indicate the number of plants. Lugoal’s staining of primary roots of wild-type Col (**M**), *lzy2;3;4* (**N**), *lzy2;3;4;pgm* (**O**), *pgm* (**P**), *sltr134-6* (**Q**), *sltr136-1* (**R**), and *sltr156-4* (**S**) at 7 days after transferring to phytocabinet. Bars indicate 100 µm.

To investigate the molecular nature of the “anti-gravitropic” phenotype in the primary roots of the *lzy2;3;4* mutant, plants were mutagenized and screened to isolate suppressor mutants. M2 seeds were obtained from mutagenized M1 populations and sown on 1× MS medium. In the first screening, plants that showed a primary root growing below the horizontal line (between 0° and −180°) were selected as *suppressor of lazy triple in root* (*sltr*) candidates (Supplemental Figure S1). We isolated 103 *sltr* candidates and obtained M3 seeds. For the second screening, we assessed the growth direction of primary roots and growth angles between the hypocotyl/root joint and root tip. Eight out of 103 *sltr* candidates changed their growth direction of the primary root. To test the reproducibility with larger populations, eight lines of M4 *sltr* seeds were sown, and we quantified their growth direction (Figure 5A–L). Although more than 50% of *lzy2;3;4* roots grew between −45° and −135° (Figure 5B), the growth direction of *lzy2;3;4;pgm* roots occurred randomly as a consequence of the *pgm* mutation, with only 25% of roots between −45° and −135° (Figure 5C). Among the eight candidates, less than 50% of *sltr4-1*, *sltr134-6*, and *sltr136-1* roots grew between −45° and −135° (Figure 5E,H,I). Since *pgm* is a *sltr* (Figure 1), the mutation causing starchless amyloplasts also may be a *sltr*. Thus, root tips of these *sltr* mutants were subjected to Lugoal’s staining to test whether *sltr* candidates were starchless. While *pgm* and *lazy2;3;4;pgm* do not accumulate starch, starch accumulation in all tested *sltr* mutants was observed (Figure 5M–S, Supplemental Figure S2). These results indicate that *sltr4-1*, *sltr134-6*, and *sltr136-1* provide new directions for future studies on the nature of the “anti-gravitropic” phenotype in primary roots.

## 3. Discussion

Overall plant architecture is determined by various environmental cues, such as light, temperature, and gravity. Previous studies have demonstrated that *LAZY1* and *LAZY1-like* family genes are involved in gravitropism in rice, *Arabidopsis*, *Medicago*, and maize [12,13,14,15,29]. *LZY1*, *LZY2*, *LZY3*, and *LZY4* facilitate gravitropism in shoots and roots, such that loss of their function is expected to cause non-responsiveness to gravi-stimulation. However, in addition to the *lzy2;3;4* and *lzy1;2;3* triple mutants losing normal gravitropism, they also exhibited “anti-gravitropic” phenotypes [14,15,16,23]. While *LAZY1* family genes have been characterized extensively in various plant species due to their effects on gravitropism, the nature of “anti-gravitropic” phenotypes of *lzy* multiple mutant had not been characterized. Here, we focused on the “anti-gravitropic” phenotypes of the *lzy1;2;3* mutant in lateral branches and the *lzy2;3;4* mutant in the primary root. 

Lateral branches and lateral roots develop at a specific angle called the gravitropic setpoint angle, GSA [30]. It has been proposed that the GSA is determined a result of the balance between gravitropism and the AGO [18,19]. Thus, we assumed that the balance between gravitropism and the AGO was disrupted in *lzy1;2;3* and *lzy2;3;4* mutants, and the AGO manifested as the “anti-gravitropic” phenotypes [24]. In the primary root, the AGO may share a similar gravity-sensing mechanism with gravitropism. Since sedimentation of starch-filled amyloplasts in collumela cells triggers gravitropsim in primary root, we investigated the contribution of amyloplasts in the “anti-gravitropic” phenotype of *lzy2;3;4* by introducing *pgm* mutation. The epistasis analysis between *lzy2;3;4* and *pgm* resulted in the suppression of “anti-gravitropic” phenotypes of *lazy2;3;4* plants by the *pgm* mutation (Figure 1B,C,F,G). Thus, based on previous studies and our results, we deduce that the gravitropism and AGO share a similar gravity-sensing mechanism in the primary root. It has been reported that a reversed asymmetric auxin distribution is formed upon gravi-stimulation in the root tip of *lzy2;3;4* [16], suggesting that this reversed auxin flow is triggered by amyloplast sedimentation. In contrast, the effect of the *pgm* mutation on lateral branches in *lzy1;2;3* plants was subtle (Figure 2C–G). Thus, it is possible that gravity-sensing mechanisms or dependency of sensing mechanisms underlying the AGO might differ between roots and shoots. Moreover, other mechanisms for gravity sensing, such as the protoplast presser hypothesis, have been proposed [31], suggesting that other mechanisms besides the starch statolith might be involved in AGO in shoots. 

It is widely accepted and supported that the endodermal cells take place in gravity sensing through the amyloplasts sedimentation in shoot. The *eal1* and *sgr1* are agravitropic mutants and commonly have defects in formation of the endodermis [8,9,10]. Thus, we introduced *eal1* and *sgr1* mutations into *lzy1;2;3* triple mutant to investigate the relation between endodermis and “anti-gravitropic” phenotype of *lzy1;2;3*. Our genetic interaction analysis demonstrates that *eal1* and *sgr1* mutations are epistatic to *lzy1;2;3*, and quadruple mutants resulted in *eal1* or *sgr1* like phenotypes (Figure 3G,H,P,Q). These results clearly indicate that functions of the *SHR* and *SCR* are required for the “anti-gravitropic” phenotype of *lzy1;2;3*. It is known that SHR and SCR proteins function together as a protein complex in endodermis development [32]. Therefore, we deduce that the endodermis plays a crucial role for AGO as well as gravitropism in shoots. 

Although *LZY1*, *LZY2*, and *LZY3* were downregulated in *eal1* and *sgr1*, a phenotypic difference between *lzy1;2;3* plants and *eal1* or *sgr1* plants was observed (Figure 3C,D,F,L,M,O). It is hard to explain the *eal1* and *sgr1* phenotypes only by downregulation of *LZY1*, *LZY2*, and *LZY3*. Thus, besides *LZY* genes, it is expected that the key component genes in AGO are altered in *eal1* and *sgr1* mutants. We have reported the difference in gene expression between wild type and *eal1* or *sgr1* in inflorescence stems and showed that *TILLER ANGLE CONTROL 1* (*TAC1*) as a markedly downregulated gene in *eal1* and *sgr1* plants [15,17]. The *TAC1* encodes a protein that shares similarity with LAZY1 family proteins but lacks the CCL domain which is essential for interacting with RLD proteins. *TAC1* was identified in rice as a regulator of tiller angle [33], and in peach tree (*Prunus persica*), *PpeTAC1* was identified as a causal gene of the *broomy* mutant that leads to vertical growth of branches [34]. In *Arabidopsis*, lateral branches of the *attac1* mutant exhibited vertical growth habits [34]. Thus, AtTAC1 might coordinate growth angle control in response to gravity signals with LZYs. However, based on the public expression database eFP browser, *AtTAC1* expression is barely detected in roots, making its contribution in AGO limited. 

To deepen our understanding of AGO in root, we isolated *lzy2;3;4* suppressors and identified three *sltr* mutants (Figure 5E,H,I). We showed that these *lzy* suppressor genes are not related to the starch biosynthetic pathway (Figure 5M–S, Supplementary Figure S2). Although further genetic and physiological analyses will be necessary, the three *sltr* mutants are promising candidates to elucidate the nature of AGO in primary roots.

## 4. Materials and Methods

### 4.1. Plant Materials and Growth Condition

*Arabidopsis thaliana* Columbia-0 (Col) was used as the wild type. *lzy1;2;3*, *pLZY1:GUS*, *pLZY2:GUS*, *pLZY3:GUS* [15], *eal1* [9], *sgr1* [7], and *pgm* [4] were previously described. *lzy2;3;4*, *lzy2;3;4;pgm*, *lzy1;2;3;pgm*, *lzy1;2;3;eal1*, and *lzy1;2;3;sgr1* were generated in this study by genetic crossing. *lzy2* and *lzy3* were previously described [15], and *lzy4* (GABI_479C08) was used. For the phenotypic analysis of primary roots, surface-sterilized seeds were sown on Murashige and Skoog medium supplemented with 1% sucrose, 0.01% myo-inositol, 0.05% MES-KOH (pH5.7), and 0.5% Gellan Gum. For germination induction, plates were kept in the dark at 4 °C for 3 days and then transferred to 22 °C in a growth chamber under continuous light (defined as Day 0). For the phenotypic analysis of axillary branches, seeds were sterilized and kept in the dark at 4 °C for 3 days, sown on soil directly, and grown under continuous light.

### 4.2. Gene Nomenclature

In this manuscript, the following genes were mentioned: *EAL1*/*SHR*/*SGR7* (At4g37650), *SGR1*/*SCR* (At3g54220), *PGM* (At5g51820), *LZY1* (At5g14090), *LZY2* (At1g17400), *LZY3* (At1g72490), *LZY4* (At1g19115), *AtTAC1* (At2g46640), *SGR5*/*IDD15* (At2g01940), *IDD14* (At1g68130), *IDD16* (At1g25250), *PIN1* (At1g73590).

### 4.3. Lugoal’s Staining

Root tips of 7-day-old seedlings were collected and incubated with 5 mM iodine solution for 1 min and rinsed with deionized water. Stained root tips were mounted in chloral hydrate solution and photographed. 

### 4.4. Shoot Inversion

After flowering, when lateral branches reached 1.5–2.0 cm, plants were inverted and photographed at 5 min intervals using the GoPro HERO Black 7. Growth angles were quantified manually from time-lapse images using ImageJ software. Experiments were repeated more than three times and representative results were used for quantification.

### 4.5. GUS Staining

Tissues were fixed in 80% ice-cold acetone for 15 min and incubated in GUS staining solution (100 mM sodium phosphate (pH7.0), 10 mM ferricyanide, 10 mM ferrocyanide, 0.1% Triton X-100, 2 mM 5-bromo-4-chloro-3-indolyl-β-D-glucuronic acid) at 37 °C. For thin section preparation, samples were fixed with 4% paraformaldehyde, dehydrated in an ethanol series, and embedded in plastic resin (Technovit 7100). Embedded samples were sectioned in 10 µm thickness with a microtome. Thin sections were stained with neutral red (0.01%) and mounted with Entellan for microscopy.

### 4.6. Data Visualization and Statistical Analysis

R (version 3.5.1) was used for data visualization and statistical analysis. The following statistical tests were used to calculate the corresponding p-values. The F-test was used to compare the distribution of two data sets. Tukey–Kramer’s test was used for multiple comparisons of all possible data pairs.

## Supplementary Materials

The following are available online at https://www.mdpi.com/2223-7747/9/5/615/s1, Figure S1: Schematic representation of genetic screening of *lzy2;3;4* suppressors. Figure S2: Lugoal’s staining of primary roots from all the *sltr* candidates. Video S1. Gravitropic response of lateral branch from wild-type Col plant; Video S2. Gravitropic response of lateral branch from *lzy1;2;3* plant; Video S3. Gravitropic response of lateral branch from *pgm* plant; Video S4. Gravitropic response of lateral branch from *lzy1;2;3;pgm*; Video S5. Gravitropic response of lateral branch from *eal1* plant; Video S6. Gravitropic response of lateral branch from *lzy1;2;3;eal1* plant.
