# Supplementary material for: Gravity-Sensing Tissues for Gravitropism Are Required for “Anti-Gravitropic” Phenotypes of lzy Multiple Mutants in Arabidopsis
Source: Plants (Basel). 2020 May 12;9(5):615. doi: 10.3390/plants9050615 (PMC7286026; doi:10.3390/plants9050615)
Supplement: Supplementary file 1 [file plants-09-00615-s001.zip › plants-774576-supplementary/plants-774576-Supplementary_Figures.docx]

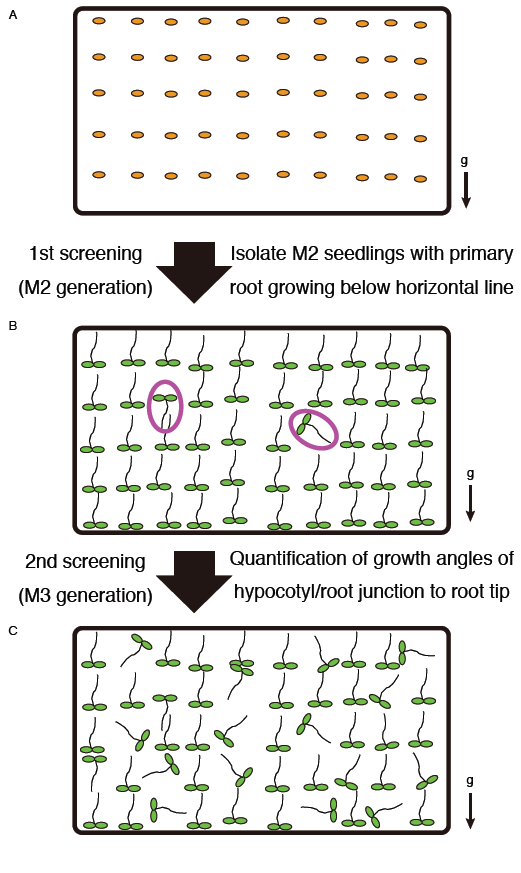


**Figure S1.** Schematic representation of genetic screening of *lzy2;3;4* suppressors. (**A**) Four hundred seeds from each M2 population were sown on MS medium (hundreds of seeds per plate)**.** (**B**) After transferring to the phytocabinet, plates were maintained in the direction of gravity for 5 days to culture M2 seedlings. Growth direction of primary roots from 5-day-old seedlings was checked, and seedlings that had primary roots growing below horizontal lines (circled in magenta) were selected as *sltr* candidates. (**C**) As a second screening, *sltr* candidates were sown on MS medium and plates were placed in the direction of gravity for 5 days to culture M3 seedlings. Growth directions of primary roots from 5-day-old seedlings were evaluated.

**
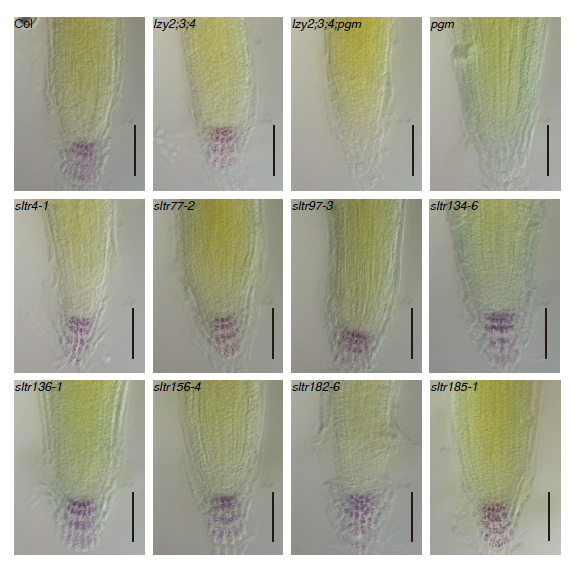
**

| **A** | **B** | **C** | **D** |
| --- | --- | --- | --- |
| **E** | **F** | **G** | **H** |
| **I** | **J** | **K** | **L** |

**Figure S2.** Lugoal’s staining of primary roots from all the *sltr* candidates. Lugoal’s staining of primary roots of Wild-type Col (**A**), *lzy2;3;4* (**B**), *lzy2;3;4;pgm* (**C**), *pgm* (**D**), *sltr4-1* (**E**), *sltr77-2* (**F**), *sltr97-3* (**G**), *sltr134-6* (**H**), *sltr136-1* (**I**), *sltr156-4* **(J**), *sltr182-6* (**K**), and *sltr185-1* (**L**) at 7 days after transferring to phytocabinet. Bars indicate 100 µm.
